# Prediction of Self-Management Behavior among Iranian Women with Type 2 Diabetes: Application of the Theory of Reasoned Action along with Self-Efficacy (ETRA)

**Published:** 2012-02-01

**Authors:** A R Didarloo, D Shojaeizadeh, R Gharaaghaji ASL, H Habibzadeh, Sh Niknami, R Pourali

**Affiliations:** 1 Department of Health and Social Medicine, Faculty of Medicine, Urmia University of Medical Sciences, Urmia, Iran; 2 Department of Health Education and Promotion, School of Health, Tehran University of Medical Sciences, Tehran, Iran; 3 Department of Epidemiology and Biostatistics, Faculty of Medicine, Urmia University of Medical Sciences, Urmia, Iran; 4 Department of Nursing, School of Nursing and Midwifery, Urmia University of Medical Sciences, Urmia, Iran; 5 Department of Health Education and Promotion, Faculty of Medical Sciences, Tarbiat Modares University, Tehran, Iran

**Keywords:** Diabetes, Self-management, Self-efficacy, Behavior, Reasoned action

## Abstract

**Background:**

Continuous performing of diabetes self-care behaviors was shown to be an effective strategy to control diabetes and to prevent or reduce its- related complications. This study aimed to investigate predictors of self-care behavior based on the extended theory of reasoned action by self efficacy (ETRA) among women with type 2 diabetes in Iran.

**Methods:**

A sample of 352 women with type 2 diabetes, referring to a Diabetes Clinic in Khoy, Iran using the nonprobability sampling was enrolled. Appropriate instruments were designed to measure the variables of interest (diabetes knowledge, personal beliefs, subjective norm, self-efficacy and behavioral intention along with self- care behaviors). Reliability and validity of the instruments using Cronbach’s alpha coefficients (the values of them were more than 0.70) and a panel of experts were tested.

**Results:**

A statistical significant correlation existed between independent constructs of proposed model and modelrelated dependent constructs, as ETRA model along with its related external factors explained 41.5% of variance of intentions and 25.3% of variance of actual behavior. Among constructs of model, self-efficacy was the strongest predictor of intentions among women with type 2 diabetes, as it lonely explained 31.3% of variance of intentions and 11.4% of variance of self-care behavior.

**Conclusion:**

The high ability of the extended theory of reasoned action with self-efficacy in forecasting and explaining diabetes mellitus self management can be a base for educational intervention. So to improve diabetes self management behavior and to control the disease, use of educational interventions based on proposed model is suggested.

## Introduction

Diabetes, as a common chronic disease, nearly in all countries is prevalent. There are an estimated 285 million adults with diabetes in 2010; this number will continue to increase globally due to an aging population, growth of population size, urbanization and high prevalence of obesity and sedentary lifestyle.[1] Iran, as one of middle-east countries, also was affected by this problem, and with regard to growth trend of elderly population size in our country, it is expected that this disease rapidly increases, and it has been reported that 2% of the Iranian population have diabetes and prevalence of diagnosed diabetes for those over the age of 30 years has estimated to be 7.3%.[2] On the other hand, this problem is gradually affecting even younger age groups, striking young adults and even adolescents.[3] Therefore, in order to prevent or delay the number of fatal complications associated with diabetes mellitus, serious glycemic control is required to achieve target blood glucose levels.[4][5] To achieve this goal, it is necessary to encourage therapeutic regimens adherence so that patients observe the medical recommendations, take their medication, change their life style, and follow the recommendations of the clinicians.[6]

Numerous studies showed that diabetes selfmanagement led to glycemic control in diabetic patients, which in turn led to better health outcomes.[4][7] Despite the scientific support for tighter control of blood sugar, many persons with diabetes did not adequately manage their condition, thus lacking good glycemic control.[8][9]

This lack of glycemic control may be a result of the lack of adherence to recommended self-management behaviors among diabetics. A number of studies have investigated the adherence rates to self-management behaviors in diabetics including medication use, insulin injections, urine and blood testing.[10][11]

As expected, results from these studies illustrate that adherence is low in most of the diabetes selfmanagement behaviors. The prevalence, associated mortality and morbidity, the economic costs, and consequently individual and the social burden of diabetes emphasize the urgent need to help diabetic patients in order to manage the disease better.[10][11]

Improving adherence to self-management behaviors is the first step towards helping patients to manage their disease better. It is important to examine and understand factors affecting self-management behaviors of diabetic patients. This will help to inform and strengthen interventions designed to improve adherence to self-management behaviors in diabetic patients. It will also help health care professionals to administer the disease better and reduce the risk of disease-related complications.[12]

There has been extensive research in trying to understand the determinants of intentions or likelihood of performing health behaviors in diabetic patients using theoretical frameworks. Typically, these studies are based on commonly used theoretical frameworks such as the health belief model (HBM), the theory of reasoned action (TRA) and the theory of planned behavior (TPB).[13][14] One of the most successful theories of behavior change is TRA created by Ajzen and Fishbein.[15] According to this theory, sociodemographic variables play an important role in determining behavior. Since behaviors that are not fully volitional are also influenced by the individual's perception of his or her ability to perform the behavior (selfefficacy) and TRA would not be sufficient to examine the relationships of health beliefs, intention is compling self-care practices in diabetic patients. Self-efficacy is defined as the perception that one can master the tasks or successfully execute the behaviors required to produce the outcomes in a given situation.[16]

Since the earliest days of the TPB, there has been a degree of uncertainty concerning the relationship between Ajzen’s perceived behavioral control and Bandura’s self efficacy construct. Both constructs concern control over the execution of the behavior including the perceived ease and difficulty of performing a behavior (perceived behavioral control) and the belief that one is capable of performing the behavior (self efficacy).[17][18] Additionally, congruent with Ajzen’s argument that self efficacy and PBC are synonymous, several researchers have incorporated measures of self efficacy within the TPB framework. Armitage and Conner suggest the existence of two processes of control, an internal and an external, discriminable but not independent of each other. According to these authors’ findings, being very confident of one’s abilities to perform a behavior may lead to underestimation of external control; likewise, the presence of external facilitating conditions that may boost self-efficacy perceptions. The lack of clarity in the conceptual and operational definitions of PBC and self-efficacy within TPB research makes it problematic to draw conclusions about which could be the most appropriate construct in this framework. Furthermore, the existence of overlap between the constructs among these theories made it difficult to compare them.[19]

On the other hand, In some studies like Tiatrakul’s study conducted on the older adults with diabetes in Thailand in 2001, it was shown that self-efficacy not only is important in managing diabetes, but also in prediction of self-care behaviors in patients with type 2 diabetes.[18] Hence it is believed that TRA along with self-efficacy may be helpful in predicting and explaining self-care of diabetics.

The proposed framework claims that intention to perform behaviors is explained by an incorporation of attitudes and subjective norms. Attitudes are beliefs that a particular behavior leads to certain outcomes, and subjective norms contain social pressures that one receives from others for engaging in a given behavior. Intentional behavior is then considered to be a function of one’s intention which is a reflection of attitudes toward the behavior and the perceived social norm.[20]

According to this theory, sociodemographic variables play an important role in determining behavior. Since behaviors that are not fully volitional are also influenced by the individual's perception of his or her ability to perform the behavior (self-efficacy), and the theory of reasoned action would not be sufficient to examine the relationships of health beliefs and intention to comply self-care practices in diabetic patients. To account for this, the TRA has been extended by adding several constructs that involve perceptions of control over behaviors.16 This study proposed an extension of the TRA that includes self-efficacy as a third determinant of behavioral intention in addition to the subjective norm and attitude toward the behavior constructs proposed in the original theory. Selfefficacy is defined as the perception that one can master the tasks or successfully execute the behaviors required to produce the outcomes in a given situation.[16] Self-efficacy beliefs reflect people's thoughts about their capability to perform certain behaviors and influence the activities that individuals choose to approach as well as their motivation and persistence in view of obstacles in performing those activities.[21]

**Fig. 1:**
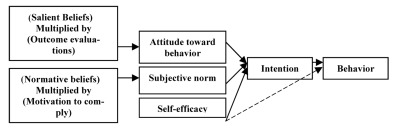
The Extended Theory of Reasoned Action applied to diabetes self- care behavior.

Most studies showed that a strong association between self efficacy and intention existed.[22] People were more likely to intend a behavior when they felt they were able to perform. The proposed model combines self-efficacy in the TRA framework as a third determinant of intention independent of attitude and subjective norm (Figure 1). This theoretical framework assumes an effect of self-efficacy on behavior that is completely mediated by intention, and that intention in turn is the immediate antecedent of the behavior. It could be argued that self-efficacy is a necessary, but not sufficient condition for the formation of intention to perform a behavior. In addition to believing that one could perform a behavior, one must be motivated to do it for other reasons. These considerations imply the possibility that self-efficacy affects intention in interaction with attitude and subjective norm. Some researches have shown that the TRA with self-efficacy (ETRA) have stronger predictive power for both intention and behavior, than the original TRA.[23][24] Wulfert et al.[24] revealed that adding self-efficacy in the TRA could improve the explaining efficacy of full model on the intention to condom use, at the same time, impeded the effects of attitude and subjective norms on behavior. Our proposed model postulates that self-efficacy may also affect behavior directly (represented by the broken line in (Figure 1). The theoretical base of this correlation is supported by Bandura’s assertion that besides the motivational component of self-efficacy, highly efficacious people are more likely to persevere in their attempts in performing a behavior in situations presenting barriers to performance attainment, while, inefficacious individuals tend to give up quickly.[16][21] Ultimately, the belief that both intention and self-efficacy are needed for the prediction of behavior and may suggest an interaction effect on behavior depending on level of perceived self-efficacy.

According to the proposed model, diabetic patients would intend to adhere to activities of self-care believing that such adherence would be associated with more positive outcomes than negative ones. If they believed that referents with whom they were motivated, they comply (physicians, spouse, family, friends and others) that they should try to adhere to such regimens. Individuals would succeed in their attempts if they had sufficient self-efficacy to master the tasks or successfully execute the behaviors required to produce the expected outcome.

On the basis of the above documents and the lack of clarity in the conceptual and operational definition of PBC construct within TPB framework, we decided to design and test a new version of TRA in order to identify predictors of self-care behavior among Iranian women with type 2 diabetes. It is expected that this might contribute to existing knowledge and help to enhance women’s health.

## Materials and Methods

The current study is a cross-sectional survey to determine predictors of self-care behavior among Iranian women with type 2 diabetes. The eligible sample consisted of 352 women with type 2 diabetes referring to the Diabetic Clinic in Khoy; Iran that was recruited for the current research. Inclusion criteria were women diagnosed with type 2 diabetes, living in Khoy, Iran, aged between 18 and 65 years, and being diagnosed as diabetic patients for at least 6 months. Participants with gestational diabetes, ulcers on the feet or progressive cardiovascular conditions were excluded.

To collect study data, questionnaires were utilized. This study was approved by Tehran University of Medical Sciences Office of Institutional Review Board.

For developing study questionnaires especially ETRA components, we utilized a manual related to constructing questionnaires based on TPB,[25] and the existing scales related to present topic in previous foreign studies.[26] The subscales concerning the theorized model were translated using a back-translation technique. Two bilingual health professionals conducted the translation into Persian. Another bilingual individual backtranslated the Persian version into English. Relevance and accuracy were checked by a doubletranslated comparison of two English versions, and necessary revisions were made. To test clarity and content validity, the Persian version was shown to a panel of experts. They evaluated each item for its distinctiveness, understandability, and appropriateness for the study’s purpose, and final revisions were made based on their comments.

Before the study, reliability of the translated survey was tested based on Cronbach’s alpha values. Cronbach’s alpha coefficients ranged from 0.70 to 0.85.

To assess levels of diabetes self-care, we used the Summary of Diabetes Self-Care Activities.[27] Respondents reported the number of days per week they performed recommended self-care activities over the past 7 days. The Cronbach’s alpha coefficient was 0.74.

Knowledge of diabetics was assessed using 11 multiple-choice questions. Each correct response was scored 2, each incorrect response, 0; and each “do not know” response, 1; with the response category ranging from 0 to 22. The subsequent results were based on the mean knowledge derived from 11 questions, each with a maximum point of 2 and a minimum point of 0. Its Cronbach’s alpha coefficient was 0.77.

The ETRA subscales included attitude toward behavior, subjective norm, self-efficacy and behavioral intention. Response categories for each item included 5-point Likert scale ranging from 1=strongly disagree to 5=strongly agree. Attitude toward diabetes selfcare activities was indirectly measured using following statements: “I think that following special diet plan to delay complications related to diabetes” and “delaying diabetes complications by following diet program is fully important for me” with Cronbach’s alpha coefficient of 0.78.

Subjective norm of diabetics was indirectly measured using the following statements: “My family supports me to participate in a suitable exercise” and “My family opinion always is important for me” with Cronbach’s alpha coefficient of 0.81.

Self-efficacy of diabetics was assessed using the following statements: “I think I am able to follow my diet when I am away from home” and “I am able to take my medicine as prescribed” and “I am able to check my blood sugar if necessary” with Cronbach’s alpha coefficient of 0.85.

Intention of diabetics was measured using the following statements: “I intend to take all my diabetes medications exactly as prescribed during the future month” and “I plan to self-monitor my blood sugar once a week during the future month” with Cronbach’s alpha coefficient of 0.74.

To determine the relative importance of demographics and ETRA variables to predict behavioral intention and actual behavior among diabetic women, we constructed two multiple linear regression models. First, we examined sociodemographics. Second, ETRA components were added to the equation to assess independent impact of ETRA controlling for other related variables. Data was analyzed using SPSS software (Version 16.0, Chicago, IL, USA). An alpha level of 0.05 was used to determine statistical significance for all analyses.

## Results

Nearly 50.9% of the participants were between 44 and 56 years old. Slightly more than half of the subjects (53%) were illiterate and also, 93.2% of them did not have any formal job except housekeepers, but the remaining (6.8%) had a part time job. About 91% of participants were currently married and approximately half of the women had a moderate monthly income. most diabetic women were overweight (46.9%) and obese (40.1%). BMI was defined according to criteria set by WHO: BMI<18.5, BMI=18.5-24.9, 25<BMI<29.9, BMI≥30 show underweight, normal weight, overweight and obesity, respectively. Most of the participants (62.2%) had diabetes for 1-10 years and approximately three-fourths of subjects (74.4%) were treated by oral hypoglycemic agents and 22.2% of cases used other therapeutic methods such as exercise, eating healthy foods, etc. and the remaining participants (3.4%) did not receive any treatment. Approximately two-thirds of the participants (63.1%) were visited by their physicians, every 1 to 2 months, and the rest, in longer term frequencies. About 78.1% of the participants did not previously participate in any of formal instructional meetings. The majority of the subjects (72.4%) were acquiring diabetes-related information from health professionals.

A factor was considered as predictor when it was significant both in bivariate analyses and in the final model of regression analysis. First, Kolmogrov Smirnov’s statistical test was used to evaluate normality of the studied variables. According to findings of the above test (normal distribution of data), we utilized parametric tests for data analysis. The t-test, and Pearson correlation coefficient indicated that significant differences were noticed between intention with education, marital status, visit by physician, knowledge level, subjective norms, self-efficacy, and attitude toward behavior (P<0.05). However self-care behavior was influenced by self-efficacy, subjective norms, attitudes, behavioral intention, knowledge, education, patients visit by physician, resource of information and participation in educational formal sessions (Table 1 and Table 2). In order to confirm the significance of the results of the bivariate analyses and explore real predictors, two models of regression analysis were also carried out on intention and behavior. In the first model, significant demographic variables in the bivariate analyses entered into a multiple linear regression equation and their effect, as independent variables, on intention and behavior was evaluated. In the regression related to intention, only patient visit by physician remained and the remaining variables were excluded from the regression equation and thereby the predictive ability of patient visit by physician was confirmed (p<0.05)(Table 3). However in the behavior-related regression analysis, knowledge , visit by physician and resource of information remained in the multiple linear regression equation and their influence was demonstrated (p<0. 01, p<0. 05, respectively)(Table 4).

**Table 1:** Univariate predictors of dependent variables of the proposed model in the current study (n=352)

****	**Behavioral Intention**			**Behavior**		
**Variables**	**Mean**±**SD**	**Student's t Test**	***P* value^a^**	**Mean**±**SD**	**Student's t Test**	***P* value**
Sociodemographic Variables						
Education		t=-3.56	0.001		t =-3.53	0.001
Illiterate	4.17±0.37			3.22±0.78		
Literate	4.32±0.38			3.52±0.83		
Marital status		t=2.03	0.04		0.06	1.86
Single	4.11±0.41			3.1±0.90		
Married	4.25±0.38			3.3±0.80		
Visit intervals of patient by physician		t=2.11	0.03		t=4.24	0.001
Once every 1-2 months	4.27±0.37			3.50±0.76		
Once More than 2 months	4.1±0.39			3.12±0.85		
Resource of obtaining information Regarding diabetes		0.83	0.4		t=-2.38	0.01
Physician and diabetes clinic personnel	4.23±0.38			3.29±0.80		
Other resource	4.27±0.39			3.52±0.85		
Participation in educational formal sessions		1.76	0.07		t=2.45	0.01
Yes	4.27±0.37			3.56±0.75		
No	4.18±0.39			3.30±0.82		

^a^ P value is related to comparison of column variable and row variable.

**Table 2:** Results of correlation matrix between independent variables and depedent variables (n=352).

**Variables **	**1**	**2**	**3**	**4**	**5**	**6**
1- Diabetes knowledge	1					
2- Attitude toward self-care	0.190	1				
3- Diabetes self-efficacy	0.053	0.571	1			
4- Subjective norms	0.018	0.508	0.498	1		
5- Behavioral intention	0.163	0.514	0.559	0.467	1	
6- Self-care behavior	0.261	0.257	0.338	0.239	0.341	1

**Table 3:** Results of multiple regression analysis on intentions (n=352)

**Independent variables**	**Beta**	**t- value**	***P *value^a^**
Model 1			
Visit of patient by physician	-0.154	-2.9	0.004
Knowledge regarding diabetes	0.107	1.9	0.06
Education	0.160	2.8	0.006
Marital status	0.088	1.7	0.092
Model 2^b^			
Visit of patient by physician	-0.118	-2.8	0.005
Knowledge regarding diabetes	0.099	2.2	0.031
Education	0.038	0.82	0.414
Marital status	0.021	0.49	0.622
Self-efficacy	0.322	6.03	0.001
Subjective norm	0.203	4.03	0.001
Attitude toward behavior	0.199	3.65	0.001

^a^ P value shows significance of regression.

^b^ Model 2: R(2)=0.416, Adjusted R(2)=0.404, F=35.020, p=0.001.

**Table 4:** Results of multiple regression analysis on self-care behavior (n=352)

**Independent variables**	**Beta**	**t- value**	***P *value^a^**
Model 1			
Visit of patient by physician	-0.272	-4.85	0.001
Knowledge regarding diabetes	0.210	3.83	0.001
Education	0.130	2.35	0.019
Participation in educational formal sessions	0.094	1.89	0.06
Resource of obtaining information regarding diabetes	-0.101	-1.98	0.048
Model 2^b^			
Visit intervals of patient by physician	-0.237	-5.16	0.001
Knowledge regarding diabetes	0.193	3.04	0.001
Education	0.068	1.27	0.205
Participation in educational formal sessions	0.074	1.56	0.118
Resource of obtaining information regarding diabetes	-0.085	-1.75	0.080
Self-efficacy	0.122	2.04	0.04
Subjective norm	0.075	1.28	0.204
Attitude toward behavior	0.015	0.239	0.811
Behavioral intention	0.209	3.95	0.001

^a^ P value shows significance of regression.

^b^ Model 2: R(2)=0.253, Adjusted R(2)=0.236, F=14.3, p=0.001.

In the second model, significant demographic variables from the previous analyses, along with variables of the hypothesized model were entered into the regression equation and their effects on the main dependent variables (intention and behavior) were examined. In the final model, patient visit by physician, knowledge, attitude, subjective norms and selfefficacy remained in the regression equation and their impact was confirmed on behavioral intention (p<0. 05)(Table 3).

However based on the results of final model of regression related to behavior, knowledge, patient visit by physician, self-efficacy and behavioral intention remained in the regression equation and their significant relationships to behavior were confirmed (p<0. 05)(Table 4).

## Discussion

In this study, we raised our understanding of diabetes self-care behavior predictors among Iranian women with type 2 diabetes, and also we determined existing gaps that needed to be addressed by future interventions. Results highlighted that significant association was visible between independent constructs of ETRA and its dependent variables, such as the presence of a significant relationship between attitudes and intention to perform self-care among diabetic women (β=0.199, p=0.001). The regression results suggest that in this women population, as attitudes towards self-management of diabetes increased, adherence to recommended diabetes self-care practices increased and led to control of diabetes. Results of study of Pattama et al. confirmed our finding about these attitudes. They demonstrated that there was a moderate positive association between attitudes and self-care (r=0.58, p<0.001).[28] Other studies[2][29] also proved the relationships between diabetes-specific health beliefs and adherence to the diabetes regimen and glycemic control in older persons with non-insulin dependent diabetes mellitus (NIDDM). Therefore, considering the study results and other aforementioned studies, we realized that attitudes play the main role in predicting intentions and diabetes self-care behavior. According to study findings, attitudes did not directly predict behavior but its effect on behavior was mediated by intention and explained 5.7% of variance of intention and was consistent with previous studies.[28][29]

Results revealed that subjective norm had a positive relationship with behavioral intention (ß=0.203, p=0.001). Results of study of Pattama et al. and Fishman et al. were consistent with our finding.[28][30] Social pressure is an important factor contributing to positive health practices. In the field of diabetes, social pressure was reported to make a significant contribution to patient's health and well-being.[31] For instance, Wang and Fenske investigated the relationship among the source of social pressure, universal selfcare, and health deviation self-care in adults with NIDDM. Significant differences were noticed between the groups with supported and the groups without support in relation to universal self-care and health-deviation self-care.[32] In other words, Subjects who received support showed a higher self-care behavior than those without support. Although, personal beliefs and subjective norms were necessary in adherence to health actions, they might not cause behavioral changes especially in behaviors of hard and complex.[21] For example, in our study, diabetic women were shown to have positive attitude to self-care behavior and intent to perform it, but due to lack of sufficient skills and abilities for engaging in a given behavior, they may not perform self-care practices. Therefore, promoting self-efficacy (SE) in diabetic patients can remove the above gap. Therefore, we found that there was a statistically positive association between self-efficacy and intention to self-care behavior (β=0.322, p=0.001). Study Results of Henrietta et al.[33] approximately were consistent with our findings. They found that self-efficacy was positively correlated with diabetes activities (r=0.470, p=0.001), indicating that more self-efficacious individuals performed more diabetes self-care activities. It was shown that self-efficacy was one of the strongest psychosocial variables related to health behavior. Greater SE was related to adhering to exercise programs,[34] quitting smoking,[35] and safer sex behavior.[36] Further, previous researchers have also found that high rates of SE enhanced adherence to diabetic control regimens.[37]

At bivariate and regression analyses, we found that every three model constructs (attitudes, subjective norms and self-efficacy) were influential on intentions and effect of them on behavior were mediated by intentions, as among these constructs, self-efficacy was the strongest predictor of intentions. Self-efficacy not only had an indirect effect on self-care behavior, but it also affected self-care behavior directly.

Investigators also measured the impact of sociodemographic factors on study dependent variables. Only patient visit by physician and diabetes knowledge, were influential on intentions and behavior. The findings revealed that there was a statistically significant difference between diabetes knowledge and self-care behavior (β=0.193, p=0.001). It means that due to increase of knowledge, probably the performance of self-care practices by diabetic women would also enhance. Moreover, diabetics' knowledge not only directly affects the self-management but also influence of knowledge on diabetes self-management was mediated by intentions (ß=0.099, p=0.031).

Results of previous studies concerning association between knowledge and diabetes self-care were consistent with our findings.[38][39][40] Theoretically, if patients are to play an active role in managing their diseases, they must be knowledgeable about their condition and its management.[38][39] Specifically, in order to manage diabetes, the individual must understand medication, diet, exercise, blood glucose self-monitoring, foot care, and how to adjust the regimen according to his/her illness situation.[39] Therefore, the relationship between diabetes knowledge and diabetes control is that increased knowledge of diabetes is correlated to improved diabetes control and that a low level of diabetes knowledge is associated with poor control.40 The literature has shown that knowledge is necessary but not sufficient to influence diabetes self-care behaviors and there are other factors affecting DMSM and glycemic control.[39] One study37 suggested that for enhancing diabetes self-management, providing adequate knowledge is important, but individuals’ beliefs and other psychosocial factors are also helpful in selfmanagement. In order to promote patients’ DMSM, educational interventions should be designed to improve knowledge and enhance beliefs of treatment effectiveness and self-efficacy.

According to current study results, patient visit by physicians is another factor affecting intentions and behavior, as a significant negative association would be noticed between that factor and intention (β=-0.118, p=0.005), and self-care behavior (β=-0.237, p=0.001). When time interval of visiting reduces, probably the performance of intention and actual behavior increases and vice versa. Visiting diabetic patients transforms a communication process between the patient and provider, under this communication, patients' knowledge regarding diabetes and management strategies and acquiring positive attitudes would increase and in turn, also intent to engage in behavior and performing diabetes self-care practices would increase. This finding of current study added support to the growing evidence showing the importance of provider-patient communication for promoting diabetes self management behaviors.[41]

Kaplan et al.[42] posits that the interaction between providers and patients reinforce patients’ confidence and therefore influence health outcomes. To facilitate patients’ adoption of diabetes self-management, concordance between providers’ beliefs and patients’ beliefs about their illnesses and treatments must be reached. Higher degree of agreement between the physician and the patient resulted in higher level of DMSM.[42]

Although study results provide important information about the relationships between DMSM and individual and environmental factors while several limitations should be considered. First, a crosssectional design was used to describe the relationship between variables. The main characteristic of crosssectional design was that all data were collected at one time period, thereby limiting the ability to identify cause and effect relationships between variables. Second, results can not be generalized beyond the study sample and therefore can be generalized only to populations with similar features. Third, the data of this study were collected using a self-reported questionnaire. Participants may underestimate or overestimate their self-management behaviors, which may affect the findings.

This research strengthened a commonly used theoretical model of health behavior change by incorporating it with an equally popular theory pressing selfevaluation. It was therefore hypothesized that the multidimensional nature of adherence could be better predicted by adding the construct of self-efficacy to the theory of reasoned action. Findings of our study showed that predictive power of ETRA was more than the original model (TRA), as ETRA model along with its related external factors explained approximately 41.6% of variance of intentions and 25.3% of variance of actual behavior. Meanwhile, among constructs of model, self-efficacy was the strongest predictor of intentions among women with type 2 diabetes, as it lonely explained 31.3% of variance of intentions and 11.4 % of variance of self-care behavior. In other words, self-efficacy not only directly predicted DMSM but also affected it indirectly. Hence, if diabetes educators promote self-efficacy of diabetics, patients would adhere regimens without feeling external obstacles. Finally, we concluded the aforementioned model to be helpful for understanding and explaining diabetes self-management behavior among Iranian women with type 2 diabetes. According to study results found among sociodemographic variables, only diabetes knowledge and patient visit by health care providers were effective on intention and behaviors and significance of them were confirmed by statistical analyses.

The results provided information for health care providers in Iran that can directly help the development of interventions aimed at improvement of diabetes selfmanagement in Iranian individuals with diabetes.

The high ability of the extended theory of reasoned action with self-efficacy in forecasting and explaining diabetes mellitus self management can be a base for educational intervention. So to improve diabetes self management behavior and to control the disease, use of educational interventions based on proposed model is suggested.
